# Oxygen Saturation Behavior by Pulse Oximetry in Female Athletes: Breaking Myths

**DOI:** 10.3390/bios11100391

**Published:** 2021-10-14

**Authors:** Pilar Martín-Escudero, Ana María Cabanas, Manuel Fuentes-Ferrer, Mercedes Galindo-Canales

**Affiliations:** 1 Professional Medical School of Physical Education and Sport, Faculty of Medicine, Universidad Complutense de Madrid, 28040 Madrid, Spain; pmartinescudero@med.ucm.es (P.M.-E.); mege@rocketmail.com (M.G.-C.); 2 Departamento de Física, Universidad de Tarapacá, Arica 1010064, Chile; 3 Unit of Clinical Management (UGC), Department of Preventive Medicine, Hospital Clínico San Carlos, 28040 Madrid, Spain; manuelenrique.fuentes@salud.madrid.org

**Keywords:** pulse oximetry, oxygen saturation, blood gas monitoring, ventilatory threshold, woman response to exercise

## Abstract

The myths surrounding women’s participation in sport have been reflected in respiratory physiology. This study aims to demonstrate that continuous monitoring of blood oxygen saturation during a maximal exercise test in female athletes is highly correlated with the determination of the second ventilatory threshold ($VT_{2}$) or anaerobic threshold (AnT). The measurements were performed using a pulse oximeter during a maximum effort test on a treadmill on a population of 27 healthy female athletes. A common behavior of the oxygen saturation evolution during the incremental exercise test characterized by a decrease in saturation before the aerobic threshold (AeT) followed by a second significant drop was observed. Decreases in peripheral oxygen saturation during physical exertion have been related to the athlete’s physical fitness condition. However, this drop should not be a limiting factor in women’s physical performance. We found statistically significant correlations between the maximum oxygen uptake and the appearance of the ventilatory thresholds ($VT_{1}$ and $VT_{2}$), the desaturation time, the total test time, and between the desaturation time and the $VT_{2}$. We observed a relationship between the desaturation time and the $VT_{2}$ appearance. Indeed, a linear regression model between the desaturation time and the $VT_{2}$ appearance can predict 80% of the values in our sample. Besides, we suggest that pulse oximetry is a simple, fairly accurate, and non-invasive technique for studying the physical condition of athletes who perform physical exertion.

## 1. Introduction

Nowadays, there has been a growing interest in studying factors affecting female physiological response to exercise, such as oxygen saturation variations [1,2,3,4,5]. However, throughout history, women have had to fight for their self-improvement in the sports world until they had the right to participate in specific sports and certain competitions like the Olympic Games [6]. The restriction was partly based on the idea that vigorous physical activity could impair women’s health and adversely affect their reproductive capacity. These myths still survive today in some countries and have limited women’s access to sports due to several factors such as social, political, religious, or biological [7,8,9,10,11,12]. Traditionally, it has been believed that the anatomical differences between men and women make men more suitable for strength sports and women for those sports that require greater flexibility [13,14,15]. However, women’s participation has reached areas previously considered exclusive to men, such as weightlifting or marathon running [16,17,18,19]. Therefore, recent decades have witnessed a remarkable expansion of women’s sport, specifically in long-duration activities [20,21,22,23,24,25], dispelling myths from other times based more on socio-cultural attitudes than on scientific research data.

Alterations in lung gas exchange occur during intense physical exercise [26,27,28,29]. These differences typically manifest as a decrease in arterial partial pressure of oxygen ($PO_{2}$), known as hypoxemia, and an associated increase in the alveolar–arterial $O_{2}$ difference (A-$aDO_{2})$, which, potentially, represents a significant barrier to endurance performance [30]. Studies of the oxygen saturation ($SO_{2}$) evolution in women during maximal exercise have shown an early decrease in oxygen saturation at markedly lower oxygen intakes than in men [31,32]. Some studies supported the controversial hypothesis that healthy active women experience a certain exercise-induced arterial hypoxia (EIAH) because of a lower vital capacity of the lung with airways of smaller diameter and smaller diffusion surface than in men of similar age, height, and body mass index (BMI), implying that the lung structure may compromise oxygen diffusion [33,34,35]. However, more recent studies performed in women in both normoxic and hyperoxic environments assert that the oxygen desaturation observed in women is the main limiting factor in achieving a higher maximum oxygen uptake, $VO_{2,max}$, levels rather than the lung size or pulmonary capacity [28,36,37,38,39,40,41].

The current progress of wearable sensors for continuous monitoring of physiological variable parameters has given evidence that using this technology to measure and quantify human responses to exercise has worthiness in improving the understanding of the exercise effects [42,43,44,45,46,47]. In particular, heart rate (HR) and oxygen saturation determination by photoplethysmography (PPG) constitute a key factor that provides relevant information to personalize training interventions [48,49,50,51,52]. The PPG sensor monitors differences in the light intensity between blood and the surrounding tissue [53,54]. These differences are associated with small variations in the tissue’s blood perfusion, providing information about the cardiovascular system, in particular, the pulse rate, oxygen saturation, blood pressure, and blood vessel stiffness [55,56]. The $SO_{2}$ in tissues is determined by optically quantifying the concentration of oxyhemoglobin and deoxy-hemoglobin [57,58,59].

Continuous monitoring by pulse oximetry is a useful, simple, fairly accurate, reproducible, and non-invasive optical technique to study oxygenation during physical effort [60,61,62,63,64,65,66,67,68]. Although it has some limitations, essentially due to cold-related deficient blood circulation [69,70], dark skin pigmentation [71,72,73], or movement artifacts [74,75,76]. Its interest does not lie in obtaining absolute values of peripheral oxygen saturation, but in the continuous recording of eventual changes. Therefore, pulse oximetry can be applied to evaluate the physical performance of different disciplines athletes adapted to the physiological responses obtaining broad correlations with oxygen uptake [59,62,77].

The aerobic threshold (AeT) represents the limit between the slight and moderate intensity of exercise (lowest intensity zone) known as the first ventilatory threshold $VT_{1}$. Exercising around $VT_{1}$ allows stimulating aerobic metabolisms while above $VT_{1}$, blood lactate and pH start to increase and decrease, respectively [78,79,80,81]. The anaerobic threshold (AnT) represents the limit between moderate and high intensity of exercise (highest intensity zone) known as the second ventilatory threshold, $VT_{2}$, which is not sustainable for a long duration [82,83]. Determining the $VT_{1}$ and $VT_{2}$ is of significant importance for both performance monitoring and training prescription because it allows us to establish different physical work zones [84,85,86,87].

This study aims to analyze the correlations between blood oxygen saturation variations and the ventilatory thresholds ($VT_{1}$ or AeT and $VT_{2}$ or AnT) during a maximum stress test in female athletes with different skin pigmentation and different physical fitness condition. Correlation analyses were carried out between the occurrence time of the $SO_{2}$ significant drops and the AeT and the AnT, the total test time, the maximum oxygen uptake, and the anthropometric variables.

## 2. Materials and Methods

### 2.1. Subjects

The criteria for subject selection were as follows: women aged from 13 to 55 performing regular practice of a competitive sport in national and regional tournaments for at least 2 years prior to the study. All subjects were trained 2 to 4 times a week between 1 and 3 h/day. The volunteers maintained this sports practice until the day before the present study was carried out. Before admittance to the study, all subjects were evaluated for their cardiovascular health. None of the volunteers reported any respiratory or cardiac disease, presenting normal spirometric values. The exercise tests were performed in the Physiology Laboratory of the Professional School of Sports Medicine of the Faculty of Medicine (Universidad Complutense de Madrid). In conformity with the review policy statement, the experimental protocol was approved by the local Ethics committee of the Hospital Clinico San Carlos (HCSC). All subjects gave written consent to participate once the procedure and the risks of the study were explained to them.

Twenty-seven active and healthy females volunteered for participation in this study. The athletes performed an incremental exercise test on a treadmill ergometer. The anthropometric characteristics, such as age, size, weight, body mass index (BMI), and skin pigmentation, are presented in Table 1.

### 2.2. Protocol and Testing Procedure

The study protocol included anamnesis with clinical and training history, physical examinations (cardiovascular and pulmonary auscultation, blood pressure, weight, and height measurements). It was followed by a maximal treadmill incremental exercise test with continuous electrocardiographic (ECG) recording, ergospirometry breath-by-breath gas analyzer (Sensor Medics Vmax Cardiopulmonary Sanro), and continuous pulse oximetry recording during warm-up, maximal exercise, and recovery using a commercial pulse oximeter (Pulsox-3i Minolta).

**Figure 1:**
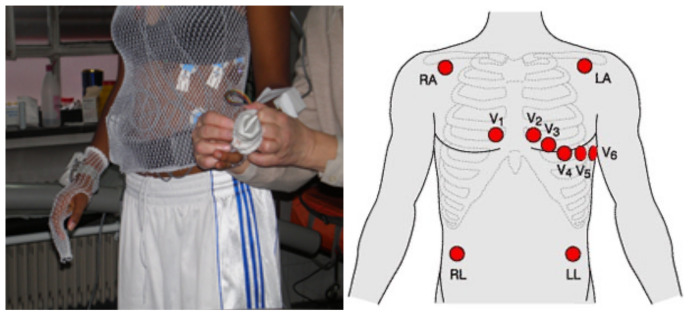
Placement of ECG electrodes in the stress test.

During the athlete preparation, 10 ECG electrodes were placed for the 12-lead EKG reading, as Figure 1 shows. The area was first prepared by shaving and alcohol sterilization to ensure a correct electrodes position wearing a tubular mesh top. Subsequently, blood pressure was taken to establish a baseline measurement, and electrocardiographic readings were taken at rest in supine and standing positions. At the beginning of the test, time and data were synchronized among ergospirometry and oximeter measurements. Parameter readings and measurements during the stress test were collected every second.

**Figure 2:**
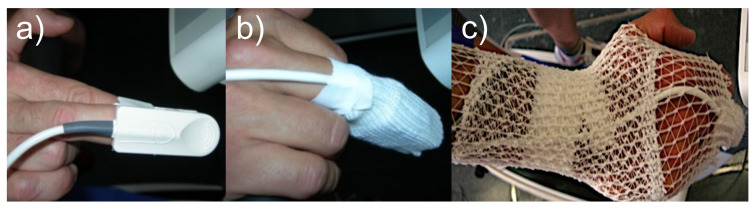
Positioning of the pulse oximeter sensor (**a**), its protection (**b**), and hand position (**c**).

Firstly, a pre-stress test forced spirometry was performed. In order to optimize the correct reading of oxygen saturation, the pulse oximeter placement area (third or fourth finger of the right hand) was cleaned with hydrophilic absorbent cotton soaked in alcohol (see Figure 2). After a minute of auto-calibration the oximetry recording started. ECG and oximeter heart rates were analyzed and compared. A sphygmomanometer was also placed in the left arm to measure blood pressure during the test.

**Figure 3:**
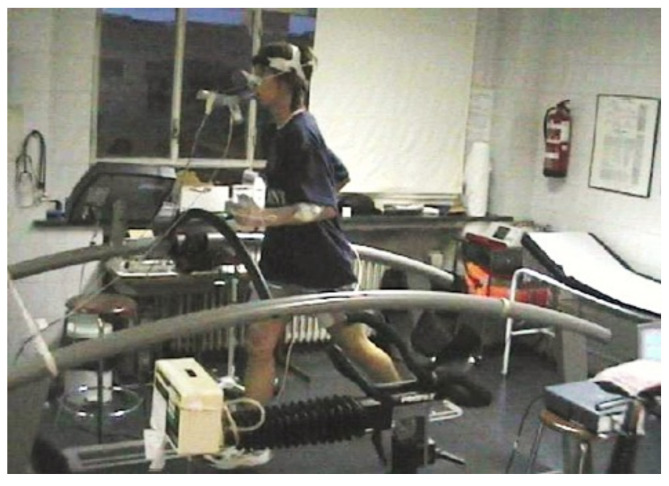
Performance athlete during the exercise stress test. The pulse oximeter is attached to the hands, the electrodes are placed for electrocardiographic recording, and the mouthpiece for gas flow analyzer.

Secondly, a mask was placed over the athlete’s nose and mouth to prevent air leakage and properly analyze expired gases, as Figure 3 shows. Before the stress test on a treadmill ergometer (HP Cosmos QUASAR 4.0) started, baseline data was collected while the athlete stood for one minute. In the warm-up, the athlete began to walk at 6 km/h and 1% slope for 2 min. The athlete started the effort phase running at 8 km/h and 1% slope. When the maximum effort condition was attained, the athlete held the protective bars and jumped off the treadmill. The maximum speed achieved varied among individuals. When the speed of 14 km/h was reached, the slope was increased to 3%. Afterward, the slope was maintained constant, while speed was increased every 2 min by 2 km/h until they could not continue. Active recovery was performed for 2 min at 8 km/h with a slope of 0%. The ECG readings were taken every 10 s, averaging the last eight heartbeats. At different test stages, once the athlete was running at fixed speed and slope, the full-step rate (SR, in steps/min) was obtained by counting the number of steps in a 10 s interval manually, and the one-foot step was derived from that. Blood pressure was taken immediately upon test completion and during the recovery period (at 3 and 5 min) by placing a sphygmomanometer in the other arm.

Every one of the athletes followed the same exercise protocol. The only difference between the tests performed by each athlete was the level of effort (stage) reached, which depended on their physical capacity. Upon the test’s completion, the oximeter and ergospirometer were disconnected.

The personal and the obtained data from ergospirometry were recorded in protocol pages and then entered into anonymized databases. Once the treadmill exercise test was performed, the ergospirometric and the commercial pulse oximeter data were compared over time by a statistical study of the variables involved.

### 2.3. Statistical Analysis

The quantitative variables were summarized in their mean, $\bar{X}$, and standard deviation (SD) for the statistical analysis. Pearson’s linear correlation coefficient and linear regression analysis were calculated to determine the relationships between the different variables. This coefficient has the property of being between +1 (perfect positive linear association) and −1 (perfect negative linear association). A null value does not indicate the absence of a relationship, but rather the absence of a linear association between the variables. The comparison between times and independent variables of two categories was performed using Student’s *t*-test for independent samples. The comparison of qualitative variables with more than two categories with quantitative variables were performed using one-factor analysis of variance (ANOVA). The time-independent effects of each of the evaluated parameters was studied through an analysis of covariance. Statistical significance was defined at the *p* < 0.05 level. All statistical analyses were performed using IBM SPSS Statistics software program version v.15.0 (SPSS Inc., Chicago, IL, USA).

## 3. Results

The results obtained from the stress test for the total population of 25 light-skinned and 2 dark-skinned healthy female athletes are shown in Table 2 and Table 3. Following the protocol described in Section 2.2, we recorded the maximum HR, the basal $VO_{2}$, the $SO_{2}$ values, and the maximum oxygen uptake, $VO_{2,max}$.

The participants were classified according to their physical fitness condition based on their maximum oxygen uptake, $VO_{2,max}$, as is shown in Table 2. The 14.8% of participants were classified by medium physical fitness condition with a $VO_{2,max}$ between 30 and 40 mL/kg/min, the 40.7% of the athletes showed a good physical condition with a consumption between 40 and 50 mL/kg/min, while the remaining 44.4% showed an excellent physical condition with a $VO_{2,max}$ greater than 50 mL/kg/min.

Since the main objective of this study was assessing whether oxygen saturation variations could be related to the AeT or AnT appearance, the $SO_{2}$ value was continuous monitored. The basal saturation was also recorded at the beginning of the test in order to analyze the differences. Table 3 shows the ergospirometry measured variables obtained from the stress test expressed by their average value ± standard deviation, $\bar{X}$ ± SD, and the average times related to the significant drops, expressed by $\bar{T}\;\pm$ SD.

The maximum HR observed ranged between values of 168 and 205 bpm, with a mean value of (189.81 ± 8.54) bpm. The basal $VO_{2,max}$ ranged from 1.7 to 7.8 mL/kg/min with a mean value of (4.69 ± 1.53) mL/kg/min. The $VO_{2,max}$ ranged from 37.09 to 64.78 mL/kg/min, with a mean value of (48.9 ± 7.61) mL/kg/min. The basal saturation ranged from 97% to 99%, with a mean value of (98.07 ± 7.61)%. To evaluate whether a higher $SO_{2}$ decrease could influence some other variables such as the AeT or AnT appearance, the $SO_{2}$ minimum value observed and the time of occurrence was recorded.

**Figure 4:**
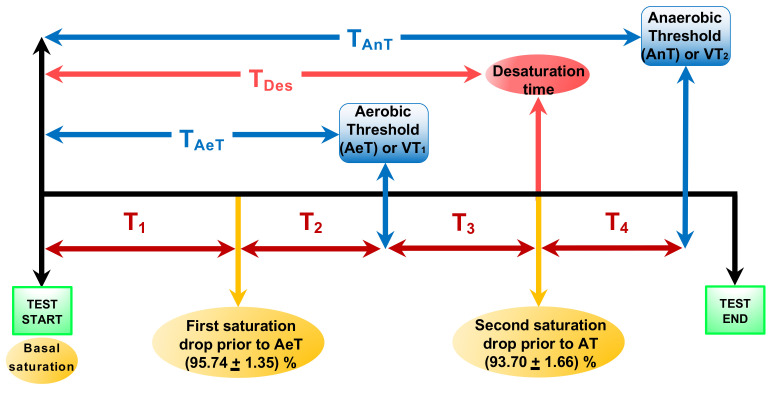
Flowchart representing the different events during the effort test. The AeT and the AnT time are marked by blue arrows. The pink arrow corresponds to the maximum desaturation time after the AeT. The time to observe the first and the second $SO_{2}$ drops are marked by the yellow arrows with the corresponding saturation values shown in the circles. The red arrows correspond to: $T_{1}$ as the time to observe the first $SO_{2}$ drop value before AeT, $T_{2}$ as the time to reach the AeT from the first $SO_{2}$ drop value, $T_{3}$ as the time to observe the second $SO_{2}$ drop value after the AeT, and $T_{4}$ as the time from the second $SO_{2}$ drop saturation to reach the AnT.

Figure 4 shows a flowchart representing the different events during the effort test. The time to observe the first and the second $SO_{2}$ drops (desaturation prior to the AeT and the maximum desaturation prior to AnT) are marked by the yellow arrows with the corresponding saturation values shown in the yellow circles. The first $SO_{2}$ drop value was (95.74±1.35)% occurring at $T_{1}=\left(3.91\pm 1.50\right)$ min. The minimum $SO_{2}$ value, (93.70±1.66)% was found between 6.28 and 10.83 min with a mean time of $T_{Des}$ = (8.97 ± 1.32) min marked by the pink arrow. We also defined $T_{2}=\left(2.15\pm 1.44\right)$ min as the time to reach the AeT from the first drop, $T_{3}=\left(2.16\pm 0.98\right)$ min as the time to observe the second $SO_{2}$ drop prior to AnT, and $T_{4}=\left(1.47\pm 1.05\right)$ min as the time to reach the AnT from the second $SO_{2}$ drop, depicted by red arrows. Besides, the blue arrows correspond to the AeT appearance time fluctuated between 4.33 and 8.17 min, with a mean value of $T_{AeT}$ = (6.06 ± 0.96) min, and the AnT appearance time ranged between 6.83 and 11.83 min, with a mean value of $T_{AnT}$ = (9.69 ± 1.20) min. The green squares correspond to the start and the end of the test. The total test time ranged between 7.83 and 14.00 min, with a mean value of $T_{Total}$ = (10.76 ± 1.45) min.

**Figure 5:**
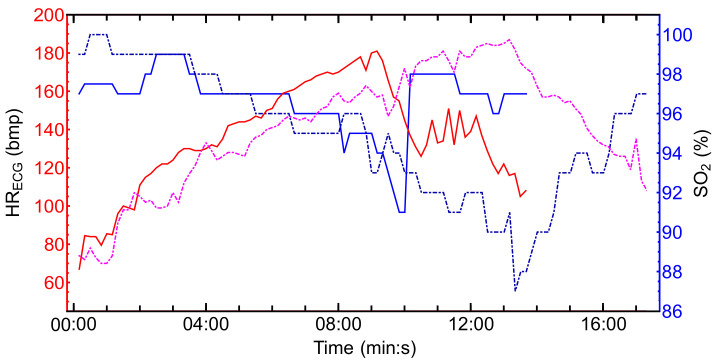
Temporal evolution of $SO_{2}$ and HR time series for two different athletes. The blue lines show the evolution of the $SO_{2}$, and the red and pink lines denote the maximum HR obtained from ECG. The continuous lines correspond to a light-skinned athlete with a medium fitness physical condition, while the dash-dotted lines correspond to a dark-skinned athlete with an excellent fitness physical condition.

A common behavior of the $SO_{2}$ evolution during the incremental exercise test was observed in all female athletes. First, we observed a $SO_{2}$ decrease before the AeT or $VT_{1}$ followed by a second significant drop before the AnT or $VT_{2}$, as it is also shown in Figure 4. The temporal evolution of the oxygen saturation $SO_{2}$ and the HR obtained from the ECG signal for two athletes is depicted in Figure 5. The blue lines correspond to the $SO_{2}$ temporal series while the red and pink lines correspond to the HR dataset in bmp. The continuous line correspond to an athlete with a medium physical fitness condition according to the criterion established in Table 2. The dash-dotted lines correspond to a dark-skinned athlete with an excellent physical fitness condition. We found several differences between both athletes. The $SO_{2}$ values range from 87% to 100% while the HR ranged from 70 bmp to 188 bmp for the dark-skinned athlete with an excellent physical fitness condition. On the contrary, for the light-skinned athlete with medium excellent physical fitness condition the $SO_{2}$ values range from 91% to 99% and the HR from 67 bmp to 181 bmp. The total test time was also longer for the athlete with a better physical fitness condition, i.e., higher $VO_{2,max}$. It can be also observed that the second saturation drop (prior to AT) occurs a few seconds after the $HR_{max}$ is reached. Once the minimum value of $SO_{2}$ is reached, the recovery phase begins. In this phase, $SO_{2}$ increases while HR decreases. Small differences were also found in the recovery time between both athletes. The recovery time, time to reach lower HR values and higher saturation values, was shorter in athletes with a better physical fitness condition.

A statistical study of Pearson’s correlations was performed concerning the evolutionary and temporal parameters of the oxygen saturation values obtained by ergospirometry. For this purpose, a univariate study was made for the different time events of the effort test shown in Figure 4 and their relationship with the study’s independent variables. Table 4 shows the Pearson’s correlation coefficients between the different time events during the effort test shown in Figure 4, i.e., AeT, the AnT, the desaturation time, total test time, and $T_{3}$ with age, height, weight, body mass index (BMI), basal $VO_{2}$, $VO_{2,max}$, and maximum HR reached during the test for each participant. The values with a significance level *p* < 0.05 are marked in bold. Statistically significant correlations were found for the maximum oxygen uptake, $VO_{2,max}$ concerning the appearance of the AeT, the AnT, the total test time, $T_{Total}$, the maximum desaturation time, $T_{Des}$, and $T_{3}$. However, no significant correlations were found between the rest of the variables and these times.

Furthermore, Table 5 shows an analysis of covariance to study the relationship between the appearance time of both thresholds (AeT and AnT), the desaturation time $T_{Des}$, the total test time $T_{Total}$, and the time to observe the second $SO_{2}$ drop value after the AeT, $T_{3}$, with respect to the skin pigmentation, physical fitness condition and type of practiced sport. The values with a significance level *p* < 0.05 are marked in bold. Statistically significant Pearson correlations were found for the physical fitness condition variable concerning the AnT occurrence, the total test time, the desaturation time, and $T_{3}$. We also found correlations between the skin pigmentation and the AnT or $VT_{2}$ appearance. However, there were no correlations for the type of practiced sport.

**Figure 6:**
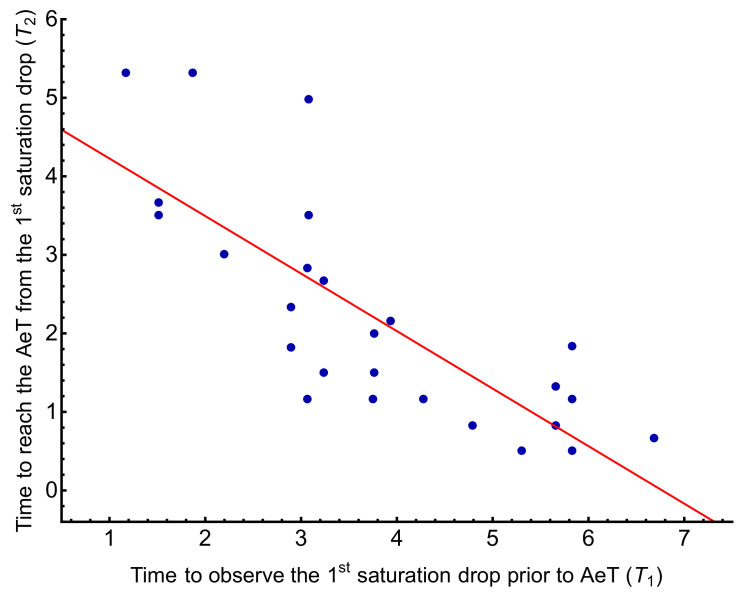
Relationship between the time of the first $SO_{2}$ drop before reaching the AeT, $T_{1}$, and the time to reach the AeT from the first $SO_{2}$ drop, $T_{2}$. The coefficient of determination is $R^{2}=0.61$ and $T_{2}=4.96-0.73T_{1}$.

In order to study the dependence of the $SO_{2}$ variations with the AeT appearance, Table 6 shows the Pearson correlations analyzed regarding the times $T_{1}$ and $T_{2}$. The values with a significance level *p* < 0.05 are marked in bold. We could not find any correlations between the AeT and these times. However, when comparing how $T_{1}$ and $T_{2}$ could influence each other, we observed a negative correlation between them with a negative Pearson coefficient of −0.789, i.e., as $T_{1}$ increases, $T_{2}$ decreases. Figure 6 shows the regression model relating both times with a coefficient of determination $R^{2}=0.61$ and $T_{2}=4.96-0.73T_{1}$, which indicates that for every minute that $T_{1}$ increases, $T_{2}$ decreases by 0.73 min.

**Figure 7:**
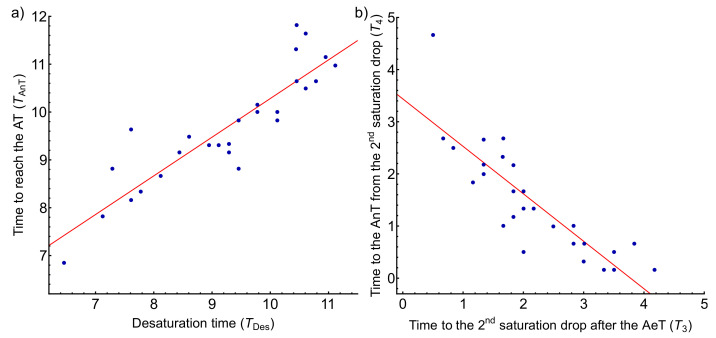
(**a**) Relationship between the desaturation time, $T_{Des}$, with the AnT appearance, $T_{AnT}$. The coefficient of determination is $R^{2}=0.80$ and $T_{AnT}=2.20+0.81T_{Des}$. (**b**) Relationship between the time to observe the second $SO_{2}$ drop after the AeT, $T_{3}$, and the time to reach the AnT from the second $SO_{2}$ drop, $T_{4}$. The coefficient of determination is $R^{2}=0.72$ and $T_{4}=3.44-0.91T_{3}$.

We proceeded in the same way to analyze the influence of the AnT appearance with the previous parameters. Table 7 shows the Pearson correlations for the AnT appearance and the times derived from the $SO_{2}$ variations occurring after the AeT. A statistically significant Pearson correlation coefficient close to 1 (0.892) when correlating the AnT appearance time with the desaturation time $T_{Des}$ is observed. The $T_{AnT}$ corresponds to $T_{1}$ + $T_{2}$ + $T_{3}$ + $T_{4}$ while the desaturation time corresponds to $T_{1}$ + $T_{2}$ + $T_{3}$, as depicted in Figure 4. Panel a of Figure 7 shows the regression model between both times with a coefficient of determination of $R^{2}=0.80$, that is, it explains 80% of the variability of the AnT as a function of the desaturation time. $T_{AnT}=2.20+0.81T_{Des}$ gives the regression line; hence it can be prophesied that for every minute that desaturation takes to appear, the AnT takes 0.81 min to appear. Furthermore, when comparing how $T_{3}$ and $T_{4}$ could influence each other, we observed a strong negative correlation between them (−0.849), i.e., as $T_{3}$ increases, $T_{4}$ decreases. Panel b of Figure 7 corresponds to the regression model between these two times with a coefficient of determination of $R^{2}=0.72$ and $T_{4}=3.44-0.91T_{3}$. The time $T_{4}$ is also correlated with the AnT appearance with a statistically significant Pearson coefficient of 0.383.

To sum up, Table 8 summarizes the most statistically significant correlations found in this study. The maximum oxygen uptake, $VO_{2,max}$, is the variable that presented the most independent correlations with each time event. However, the variables with the highest correlations were the desaturation time, $T_{Des}$, and the appearance of the AnT.

## 4. Discussion

The athletes subjected to the study practiced different multi-sprint-based sports. In particular, 14 volunteers practiced 11-a-side football (aerobic-anaerobic sport), 10 long-distance athletics (aerobic sport), 1 sprint athletics (anaerobic sport), and 2 basketball (aerobic–anaerobic sport). Therefore, 18.51% of the sample practiced aerobic-type sports, 22.2% practiced anaerobic-type sports, and 59.2% practiced mixed-type sports.

When comparing the validity of the population sample size with other similar studies, it turned out to be positive. In particular, the study by Harms [31] was conducted on a sample of 29 women, the study by St. Croix et al. [34] included 28 women. The sample size was 27 in more recent studies such as Wetter et al. [88], and even smaller in the one by Yoshiga and Higuchi who tested 16 women [89] and the performed by Zavorsky et al. and Woorons et al. [90] with only 14 females [91].

According to the physical fitness condition, although we found a study that specified the maximum oxygen uptake [31], the vast majority of the studies did not specify a quantitative scale to classify the physical fitness condition of the sample population. In these studies, the authors refer to a very varied physical level from sedentary to more trained people with maximum oxygen uptake in a range between 31 and 70 mL/kg/min [34,88,89,90,91]. A performed study in men and women mountaineering trainees at 4350 m above sea level showed that the reduction of maximum aerobic capacity was lower in women than in men under similar modes of ascent [92]. On the other hand, recent studies have shown that women tend to perform better in long-duration activities such as marathons, ultra-cycling, or long-distance swimming [22,23,24]. Taking this into account, it is striking that some studies attribute the presence of decreases in peripheral oxygen saturation in women to inconclusive problems of the respiratory system’s inadequacy to physical exertion. It should be pointed out that oxygen consumption is not a parameter that defines lung function, but instead helps to determine an individual’s physical fitness condition. Therefore, studies that rely on this parameter to check lung function can lead to erroneous conclusions. Detailed sex comparisons are difficult because the number of subjects studied to date has been woefully small, and more subjects are needed to be tested to confirm the hypothesis mentioned above [27,40,41,92,93,94,95].

Although there is evidence that hypoventilation may play a role in decreased pulmonary gas exchange in women during exercise, it appears that ventilation cannot fully compensate for the increased *A*-$aDO_{2}$ [35,91,93]. Other studies assert that acute ventilatory response to hypoxia (AHVR) is not related to the development of EIAH during maximal exercise in trained endurance and untrained individuals (men or woman) [29,96,97]. Indeed, concerning the degree of EIAH at the onset of high-intensity exercise, prolonging exercise to exhaustion had no other deleterious effects on *A*-$aDO_{2}$, and the degree of gas exchange impairment was not related to individual differences in small or large airway function or reactivity [88]. The AHVR was related to the peak of oxygen consumption, but not to oxygen saturation. Oxyhemoglobin saturation $SO_{2}$ values were lower in trained men (91.4±0.9)% and women (91.3±0.9)% compared to untrained men and women (94.4±0.8)% vs. (94.3±0.7)%, respectively [96]. Trained female cyclists demonstrated EIAH to the same degree as trained male cyclists and that some individual untrained females who also exhibited EIAH [97,98]. On the other hand, other studies have shown that athletes training for endurance sports can produce arterial blood desaturation during exercise at sea level [88,99,100]. These observations support the idea that, among the limiting factors of maximal oxygen uptake, in addition to the arteriovenous difference, *A*-$aDO_{2}$, the role of hemoglobin oxygen saturation should be considered.

Other studies describe a $SO_{2}$ decrease at maximum effort and an apparent increase in the desaturation time, $T_{Des}$, proportional to the $VO_{2,max}$ [49,60,101]. For example, the highest $VO_{2,max}$ values reported in trained subjects were obtained in individuals engaged in endurance modalities with values above 75 mL/kg/min [102]. However, values below 65 mL/kg/min for females are easily detected in high-level athletes in endurance competitions, while sedentary female subjects are closer to values of 40 mL/kg/min. These data are broadly coincident with those reported in our study, in which $VO_{2,max}$ sorted the physical condition of the athletes. Besides, regarding the maximum HR reached during the test, we found a difference from our study with a mean value of (189.81 ± 8.54) bpm (ranging from 168 to 205 bpm) when compared with the results of Yoshiga and Higuchi [89], who obtained a mean value of (196 ± 8) bpm or Lauresen et al. who measured a mean of (199 ± 5) bmp [103]. This difference can be associated with our sample being competitive athletes, who have a lower HR due to adaptations that exercise produces on the cardiovascular system.

In this framework, pulse oximetry is an appropriate method for determining the limit of cardiopulmonary stress in exercise testing characterized by a significant drop in oxygen saturation value [63,68,104,105]. Therefore, its application may be extended to the medical clinic to continuously monitor arterial blood oxygenation during exertion in people with known diseases [106,107,108,109,110] and at high altitudes [39,111,112,113,114,115].

### 4.1. Analysis of Correlations between Stress Test and Transcutaneous Oximetry

Correlations were sought for the different time events of the effort test with the variables from ergospirometry and anthropometric variables such as age, height, weight, BMI, basal $VO_{2}$, $VO_{2,max}$, and maximum HR as shown in Table 4. We only found a statistically significant Pearson correlation concerning the $VO_{2,max}$ between the different times. Furthermore, when comparing the same time events with respect to the skin pigmentation, physical fitness condition, and type of practiced sport variables (see Table 5), we found correlations for the physical fitness condition, classified based on the $VO_{2,max}$. In the case of the desaturation time, $T_{Des}$, and the total test time, $T_{Total}$, we found that the better the physical condition, the longer it takes to reach desaturation and the longer the total test time. When comparing the three levels of physical fitness, significant differences were observed between the medium and good level and the medium and excellent, with no differences between the good and excellent.

Regarding correlations between the anthropometric variables, although some studies have found a weak negative correlation for BMI and $VO_{2,max}$ [116,117] in healthy adults, other studies performed in healthy boys did not find any significant correlations [118]. Our results do not show any correlations with BMI, or any other anthropometric variable as size, weight or age as it is shown in Table 4.

### 4.2. Analysis of Correlations between the Time of Occurrence of Oxygen Desaturation, $T_{Des}$, and the Appearance of the Aerobic and Anaerobic Thresholds, $Vt_{1}$ and $Vt_{2}$

A common tendency in the $SO_{2}$ evolution during the effort test characterized by two drops before and after the AeT was observed in all athletes was observed. These findings agree with some other authors’ observations who found equivalent oxygen desaturation in males with $VO_{2,max}>60$ mL/kg/min and females with $VO_{2,max}$ with 40–55 mL/kg/min [31,32]. In our case, we observed a maximum of 7% desaturations in all-female athletes with a $VO_{2,max}$ ranged from 37 to 64 mL/kg/min.

Table 4 shows a Pearson coefficient of 0.469 between the $VO_{2,max}$ and the AeT appearance. We also compared the AeT appearance time with the skin pigmentation, the type of practiced sport (aerobic, anaerobic, or mixed), and the physical fitness condition. Statistical significance was only found when comparing the different levels of physical fitness condition based on the maximum oxygen uptake, as shown in Table 5.

To study the $SO_{2}$ variations as a factor associated with the appearance of the AeT we analyzed the time to observe the first $SO_{2}$ drop before the AeT, $T_{1}$, and the time from reaching this minimum value until the AeT appearance, $T_{2}$ (see Figure 4). A possible relationship between these two times and the AeT appearance was sought by calculating Pearson’s linear correlation coefficient and linear regression. No correlations were found between $T_{1}$ and $T_{2}$ with the AeT appearance. However, we found a negative correlation between $T_{1}$ and $T_{2}$ with a Pearson’s coefficient of −0.789, with $T_{2}$ decreasing as $T_{1}$ increases by $T_{2}=4.96-0.73T_{1}$, as shown in Figure 6.

Few studies have made comparisons between the AeT appearance and its relationship with oxygen saturation. For example, Gaston et al. investigate the impact of EIAH developed at sea level on exercise responses at moderate acute altitude in biathletes [99] based on the continuous monitoring of $VO_{2,max}$, $SO_{2}$, and $HR_{max}$. Besides, the occurrence of EIAH at sea level was associated with specific muscle and cerebral oxygenation responses to exercise under both normoxia and moderate hypoxia in [111], but a relationship with the AeT was not established.

Furthermore, we also studied $SO_{2}$ variations as a factor associated with the AnT appearance and the times $T_{3}$ and $T_{4}$ by calculating Pearson’s linear correlation coefficient and linear regression. We found a weak correlation with $T_{4}$ showing a Pearson coefficient of 0.383. However, the most significant result was obtained when correlating the AnT appearance time ($T_{1}$ + $T_{2}$ + $T_{3}$ + $T_{4}$) with the desaturation time ($T_{1}$ + $T_{2}$ + $T_{3}$), with a statistically significant Pearson correlation of 0.892. Panel a of Figure 7 shows the regression model between these two times fitted by the line $T_{AnT}=2.20+0.81T_{Des}$ with a coefficient of determination of $R^{2}=0.80$. Hence, it can be prophesied that for every minute that desaturation takes to appear, the AnT takes 0.81 min to appear.

A statistically significant Pearson correlation of 0.671 was found between AnT and the maximal oxygen uptake, $VO_{2,max}$, as shown in Table 4. This correlation seems logical, clearly explaining that an athlete with a better physical condition (higher $VO_{2,max}$) takes longer to reach the AnT. Furthermore, regarding Table 5, we also found correlations between AnT and the skin pigmentation variable. We found we found that dark-skinned athletes took an average of 1 min 46 s longer to reach the AnT. This difference may be attributed to dark-skinned female athletes were in better physical condition than light-skinned female athletes. However, these results are not representative since the sample size of dark-skinned female athletes was relatively small (*N* = 2).

In summary, concerning the influence on the appearance of the AnT, it seems clear that desaturation time plays an important role. We also found a weak correlation with $T_{4}$, which agrees with the study of Nikooie et al. who found statistically significant correlations between the desaturation time, $T_{Des}$, and the appearance of the AnT or $VT_{2}$ [119].

## 5. Conclusions

Transcutaneous oximetry during physical exertion allows more generalized and frequent monitoring of oxygen saturation evolution at peripheral level and the physical performance evaluation of the athletes. This evaluation can be perfectly adapted to the characteristics of each sport, obtaining broad correlations with maximum oxygen uptake. Our results reaffirm the importance of the use of pulse oximetry as an appropriate method for determining the limit of cardiopulmonary stress, characterized by a significant drop in oxygen saturation values.

We observed a common behavior of the transcutaneous oxygen saturation evolution during the incremental exercise test in all female athletes. First, a $SO_{2}$ decrease before the AeT followed by a second significant drop between the AeT and the AnT. Statistically significant correlations were found between the ergospirometry measured variables obtained from the stress test and the different time events related to the saturation drops. Table 8 summarizes the most statistically significant correlations found in this study. The maximum oxygen uptake, $VO_{2,max}$, is the variable that presented the most independent correlations with each time event. However, there is no evidence of a relationship between a higher decrease in oxygen saturation with the $VO_{2,max}$, which seems to indicate that the athlete’s physical fitness condition does not depend on the magnitude of the oxygen desaturation, but instead with the time to reach the maximum desaturation, $T_{Des}$.

Correlations were found between the times related to the AeT or first ventilatory threshold, $T_{1}$ and $T_{2}$, with a linear dependence given by $T_{2}=4.96-0.73T_{1}$. Besides, the times related to the AnT or second ventilatory threshold, $T_{3}$ and $T_{4}$, were also correlated following a linear relationship fitted by $T_{4}=3.44-0.91T_{3}$.

The highest correlations were found between the desaturation time, $T_{Des}$, and the appearance of the AnT. The linear regression model of the $T_{Des}$ with the AnT appearance time in female athletes predicts that for every minute that desaturation takes to appear, the AnT takes 0.81 min ($T_{AnT}=2.20+0.81T_{Des}$) and predicts the 80% of the values ($R^{2}=0.80$), as shown in Figure 7. This result is of great interest because it indicates the oxygen saturation value as an indicator with future potential in determining the second ventilatory threshold, $VT_{2}$. Besides, the AnT appearance is also correlated with the physical condition level, which suggests that the physiological cause of the oxygen decrease saturation in female athletes is the level of the physical fitness condition. Therefore, it should not represent a limiting factor of physical performance in women. Despite increasing evidence in sex-based differences in respiratory physiology, a holistic understanding of the impact on the respiratory response to exercise remains incomplete and research in a more heterogeneous population is required.

## Figures and Tables

**Table 1:** Skin pigmentation and anthropometric characteristics of the population studied. Values are expressed as mean ± standard deviation, $\bar{X}$± SD.

		Dark Skin	Light Skin	Total
N		2	25	27
	**Age (years)**	**Size (cm)**	**Weight (kg)**	**BMI (kg/m${}^{2}$)**
**$\bar{X}\;\pm$ SD**	(22.96 ± 6.19)	(163.81 ± 6.90)	(57.24 ± 6.70)	(21.31 ± 1.98)
Minimum	14	155	41.7	16.7
Maximum	39	182	75.4	25.18

**Table 2:** Descriptive variables of the population according to physical fitness condition.

Physical Fitness Condition	${\mathrm{VO}}_{2,\max}$	Frequency	Percentage
Medium	30–40 mL/kg/min	4	14.8%
Good	40–50 mL/kg/min	11	40.7%
Excellent	>50 mL/kg/min	12	44.4%

**Table 3:** Ergospirometry measured variables obtained from the stress test and average time for each event expressed by their mean ± standard deviation, $\bar{X}$± SD.

	$\bar{X}\;\pm$ SD		$\bar{T}\;\pm$ SD
HR max	(189.81 ± 8.54) bmp	$T_{1}$	(3.91 ± 1.50) min
Basal $VO_{2}$	(4.69 ±1.53) mL/kg/min	$T_{2}$	(2.15 ± 1.44) min
$VO_{2,max}$	(48.90 ± 7.62) mL/kg/min	$T_{3}$	(2.16 ± 0.98) min
Basal $SO_{2}$ value	(98.07 ± 0.616)%	$T_{Des}$	(8.97 ± 1.32) min
First $SO_{2}$ drop value	(95.74 ± 1.35)%	$T_{4}$	(1.47 ± 1.05) min
		$T_{AeT}$	(6.06 ± 0.96) min
Second $SO_{2}$ drop value	(93.70 ± 1.66)%	$T_{AnT}$	(9.69 ± 1.20) min
		$T_{Total}$	(10.76 ± 1.45) min

**Table 4:** Pearson’s correlation coefficients between the different time events during the effort test shown in Figure 4 and the anthropometric and ergospirometry variables. Values with a significance level *p* < 0.05 are marked in bold.

	$T_{\mathrm{AeT}}$	$T_{\mathrm{AnT}}$	$T_{\mathrm{Des}}$	$T_{\mathrm{Total}}$	$T_{3}$
Age (years)	−0.068	0.036	0.176	0.024	0.126
Size (cm)	0.200	0.317	0.313	0.307	0.239
Weight (kg)	0.010	0.031	0.187	−0.079	0.054
BMI (kg/m${}^{2}$)	−0.168	−0.24	−0.038	−0.365	−0.138
$VO_{2}$ basal (mL/kg/min)	0.096	0.025	0.050	0.132	0.083
$VO_{2,max}$ (mL/kg/min)	0.469	0.671	0.466	0.620	0.500
HR max (bmp)	−0.039	−0.190	−0.157	−0.201	−0.058

**Table 5:** Correlations between the AeT and AnT appearance, $T_{Des}$, $T_{Total}$, and $T_{3}$ concerning the skin pigmentation, the physical fitness condition and the type of sport practiced expressed by $\bar{X}\;\pm$ SD. Values with a significance level *p* < 0.05 are marked in bold.

	$T_{\mathrm{AeT}}$	$T_{\mathrm{AnT}}$	$T_{\mathrm{Des}}$	$T_{\mathrm{Total}}$	$T_{3}$
Skin pigmentation					
Light skin	5.97 ± 0.94	**9.56** ± **1.14**	8.84 ± 1.28	10.65 ± 1.42	9.91 ± 1.62
Dark skin	7.16 ± 0.23	**11.33** ± **0.47**	10.58 ± 0.35	12.08 ± 1.53	11.75 ± 1.06
Physical fitness					
Excellent	6.13 ± 0.82	**10.11** ± **1.08**	**9.08** ± **1.24**	**11.29** ± **1.22**	**10.44** ± **1.59**
Good	6.34 ± 1.00	**9.84** ± **0.88**	**9.51** ± **0.94**	**10.87** ± **1.36**	**10.48** ± **1.12**
Medium	5.04 ± 0.63	**8.00** ± **0.96**	**7.12** ± **0.84**	**8.83** ± **0.72**	**7.66** ± **1.03**
Type of practiced sport					
Aerobic	5.80 ± 0.61	9.90 ± 1.09	8.73 ± 1.03	10.80 ± 1.18	9.40 ± 0.71
Anaerobic	6.25 ± 0.84	10.00 ± 1.05	9.22 ± 1.43	11.19 ± 1.32	10.72 ± 1.89
Mixed	6.07 ± 1.10	9.51 ± 1.30	8.94 ± 1.41	10.58 ± 1.61	1.000 ± 1.74

**Table 6:** Pearson correlation and significance level between $T_{AeT}$ and the times derived from the stress test. The values with a significance level *p* < 0.05 are marked in bold.

	$T_{\mathrm{AeT}}$	$T_{1}$	$T_{2}$
$T_{AeT}$	1	0.375	0.274
$T_{1}$	0.375	1	**−0.789**
$T_{2}$	0.274	**−0.789**	1

**Table 7:** Pearson correlation and significance level between $T_{AeT}$ and variables derived from $SO_{2}$ data. The values with a significance level *p* < 0.05 are marked in bold.

	$T_{\mathrm{AnT}}$	$T_{3}$	$T_{4}$	$T_{\mathrm{Des}}$
$T_{AnT}$	1	−0.055	**0.383**	**0.892**
$T_{3}$	−0.055	1	**−0.849**	−0.014
$T_{4}$	**0.383**	**−0.849**	1	0.264
$T_{Des}$	**0.892**	−0.014	0.264	1

**Table 8:** Pearson’s coefficients of most statistically significant correlated variables obtained from the stress test.

Correlated Variables	Pearson’s Coefficients
$VO_{2,max}$ and total test time	**0.620**
$VO_{2,max}$ and desaturation time	**0.466**
$VO_{2,max}$ and $T_{AeT}$	**0.469**
$VO_{2,max}$ and $T_{AnT}$	**0.671**
$T_{1}$ and $T_{2}$	**−0.789**
$T_{3}$ and $T_{4}$	**−0.849**
$T_{Des}$ and $T_{AnT}$	**0.892**

## Abbreviations

The following abbreviations are used in this manuscript:

AeT	Aerobic threshold
AnT	Anerobic threshold
$VT_{1}$	Fist ventilatory threshold
$VT_{2}$	Second ventilatory threshold
$SO_{2}$	Oxygen saturation
$VO_{2,max}$	Maximal oxygen uptake
HR	Hear rate
BMI	Body mass index
EIAH	Exercise-induced arterial hypoxemia
AHVR	acute hypoxia ventilatory response
$T_{AeT}$	Time to reach the aerobic threshold
$T_{AnT}$	Time to reach the anaerobic threshold
$T_{Des}$	Desaturation time
$T_{Total}$	Total test time
$T_{1}$	Time to observe the first saturation drop value before AeT
$T_{2}$	Time to reach the AeT from the first saturation drop value
$T_{3}$	Time to observe the second saturation drop value after the AeT
$T_{4}$	Time from the second saturation drop saturation observed to reach the AnT
$R^{2}$	Coefficient of determination
PPG	Photoplethysmography
$PO_{2}$	Partial pressure of oxygen
*A*-$aDO_{2}$	Alveolar-arterial $O_{2}$ difference
